# Design and optimization of three-dimensional composite multilayer cylindrical pentamode metamaterials for controlling low frequency acoustic waves

**DOI:** 10.1038/s41598-022-09313-7

**Published:** 2022-04-04

**Authors:** Chengxin Cai, Xue Wang, Qifu Wang, Mingxing Li, Guangchen He, Zhaohong Wang, Yao Qin

**Affiliations:** 1 Key Laboratory of Grain Information Processing and Control (Henan University of Technology), Ministry of Education, Zhengzhou, 450001 China; 2 College of Information Science and Engineering, Henan University of Technology, Zhengzhou, 450001 China; 3 Institute of Applied Physics, Henan Academy of Sciences, Zhengzhou, 450001 China; 4 Key Laboratory for Physical Electronics and Devices of the Ministry of Education, Xi’an Jiaotong University, Xi’an, 710049 China

**Keywords:** Acoustics, Mechanical properties

## Abstract

For three-dimensional pentamode metamaterials, it is of great significance to realize underwater low frequency acoustic wave control. Therefore, in order to compare with traditional double-cone pentamode metamaterials, two multilayer composite cylindrical three-dimensional pentamode metamaterials with low frequency and broad band gaps are proposed in this paper. By using pentamode metamaterials with lattice constants on the order of centimeters, the phononic band gaps below 60 Hz and the single-mode area below 30 Hz can be obtained. In addition, compared with asymmetrical double-cone locally resonant pentamode metamaterials, the lower edge frequency, relative bandwidth and figure of merit of the first phononic band gap can be reduced by up to 61.4%, 10.3% and 40.6%, respectively. It will provide reference and guidance for the engineering application of pentamode metamaterials in controlling the ultra-low frequency broadband acoustic waves, vibration and noise reduction.

## Introduction

The concept of pentamode metamaterials (PMs) was first proposed by Milton and Cherkaev in 1995^1^. The equivalent elastic properties of PMs can be expressed as in the six-dimensional stress space, only the eigenvalue of the volume compression mode is not zero, and the other five characteristic values of the shear mode are zero. Such a solid structure will show the mechanical properties of traditional fluids as a whole, it is a complex “fluid” with solid characteristics. In theory, the acoustic metamaterials composed of such a periodic arrangement of structural units can achieve a perfect match with water. Therefore, the characteristics of adjustable modulus anisotropy, solid characteristics and wide frequency endow the PMs with excellent acoustic waves control capabilities, which have important potential applications in the fields of acoustic waves control, earthquake protection, vibration and noise reduction^2–12^.

In 2006, Milton et al*.* introduced the feasibility of using PMs for elastic wave cloak through the change law of traditional elastic dynamics equations under curve transformation^15^. In 2007, Chen et al*.* used the invariance of the direct current conductance equation under coordinate transformation to establish a one-to-one correspondence between the acoustic waves equation and the direct current conductance equation, and derived the general three-dimensional transformation acoustic equation for the first time^16^. In 2008, Norris systematically analyzed the inertial acoustic cloak and the PMs acoustic cloak, and proposed the possibility of applying the transform acoustic theory to the PMs^2^. In 2010, Scandrett et al*.* proposed a method of designing acoustic cloaks using layered PMs^17,18^. In 2012, Kadic et al*.* used laser direct writing technology and artificial additive manufacturing technology to produce three-dimensional PMs samples of micrometer and millimeter order for the first time, and conducted mechanical and acoustic properties research^19,20^. In 2014, Aravations-Zafiris et al*.* designed a three-dimensional layered column structure with pentamode characteristic, which broadened the array structure of PMs units^21^. This has also aroused the interest of researchers in the different lattice configurations of PMs^22^. In 2018, Chen et al*.* designed a carpet-style PMs acoustic cloak suitable for underwater broad by adjusting the microstructure geometric parameters of the two-dimensional PMs, provided a new choice for PMs in acoustic cloak devices^10^. In 2021, Quadrelli et al*.* designed, fabricated and experimentally validated a pentamode cloak for scattered power reduction in underwater acoustics by adopting a linear quasi-symmetric map defined in elliptic coordinates^13^. Cushing et al*.* experimentally verified anisotropic sound speeds predicted by finite element simulations using additively manufactured anisotropic three-dimensional PMs sample made of titanium and gave a method for extracting the longitudinal PMs wave speed based on Fourier series expansions for frequency domain simulations^14^.

So far, the majority of the studies are based on the design of the traditional double-cone structure. Through the introduction of geometric perturbations^24,25^, structural anisotropies^26^, diverse lattice types^22^ and locally resonance units^27^, single-mode properties, figures of merit and phononic band gap (PBG) of three-dimensional PMs have been studied. However, the performance of the double-cone PMs is limited due to the limited tunable structure parameters, large connection overlap deviation^28^ and the realization for ultra-low frequency acoustic waves control.

At present, low-frequency detection sonars with operating frequencies below 300 Hz have appeared, which requires PMs to have the ability to control underwater low-frequency acoustic waves. For the PMs composed of simple materials, they generally belong to the Bragg scattering PMs. If the Bragg scattering PMs is used to control low-frequency acoustic waves below 300 Hz, the structural size of the acoustic waves control device needs to be at least tens of meters, which brings great difficulties in engineering application. Therefore, studying the method and mechanism of small-scale PMs to control underwater low-frequency acoustic waves and establishing a three-dimensional control method for working frequency bands are the core issues that need to be solved urgently. Herein, we propose two cylindrical three-dimensional locally resonance PMs with ultra-low frequency and broad band gaps. The primitive of multilayer composite cylindrical is composed of composite materials, one part provides the quality required by the structural unit, that is, a hard material. The other part provides the elasticity required by the resonance of the structural unit, that is, a soft material. Furthermore, in order to compare with the traditional double-cone locally resonant PMs, the finite element simulation software COMSOL Multiphysics is used to calculate and compare their band structure, single-mode properties and pentamodal performance. Furtherly, the effects of asymmetry degrees on the PBG, single-mode properties and figure of merit (FOM) of the three samples are discussed. The lower frequency and broad band gaps will endow the proposed structure with better potential for underwater acoustic waves control.

## Structure design and energy band characteristics of multilayer composite cylindrical three-dimensional PMs

**Figure 1:**
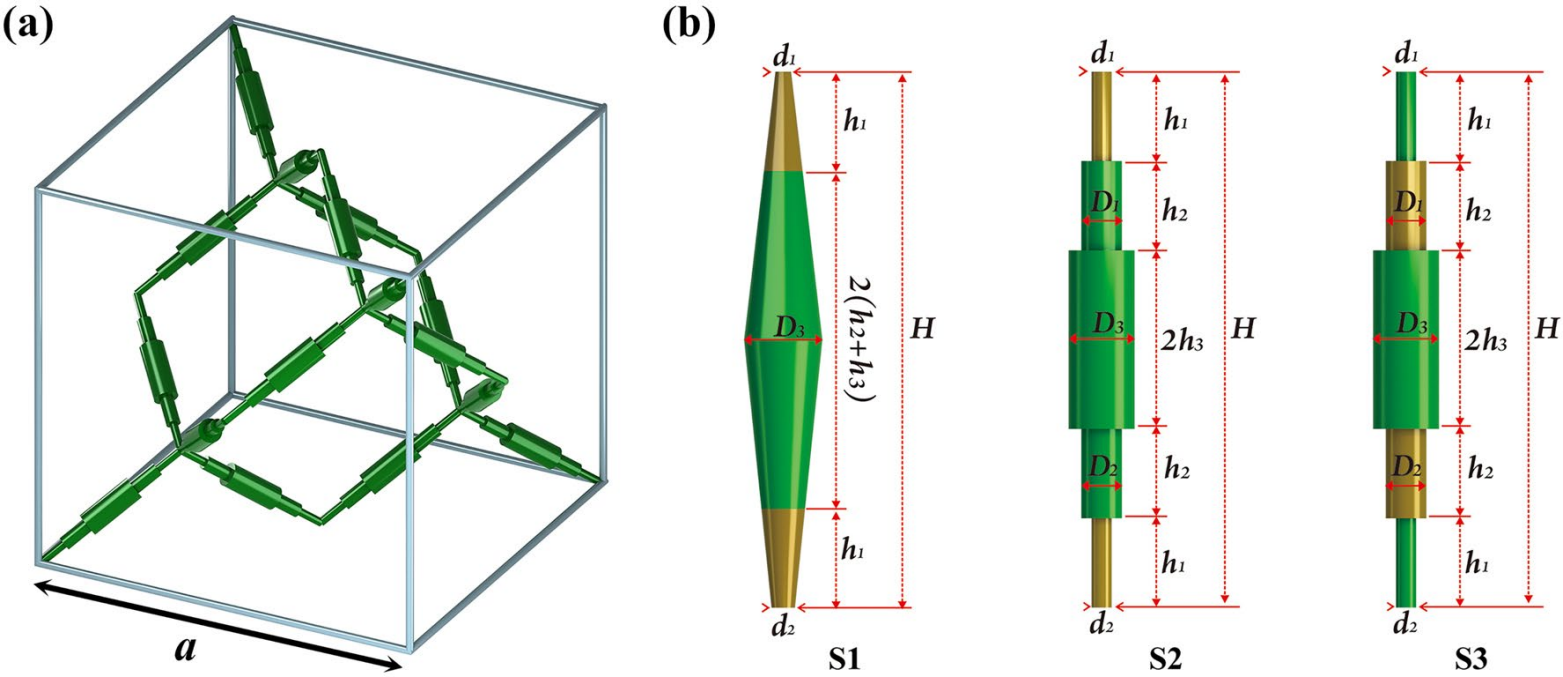
(**a**) The unit cell structure diagram of the multilayer composite cylindrical three-dimensional PMs. (**b**) Primitive structure diagrams of three locally resonance PMs.

The unit cell structure of multilayer composite cylindrical three-dimensional PMs is shown in Fig. 1a. It consists of 16 primitives connected at the narrow end to form a face-centered cubic structure with a lattice constant of *a*. Three types primitives are shown in Fig. 1b and composed by two materials. In order to comparative analysis conveniently, we define them as S1, S2 and S3, respectively. For S1, the primitive structure is the composite asymmetric double-cone element. The soft material is at the narrow diameter at both ends (${d}_{1} \mathrm{and} {d}_{2}$), and the length is ${h}_{1}$. For S2, it is one of multilayer composite cylindrical three-dimensional PMs. The soft material is added at both ends, the length is ${h}_{1}$, and the diameters from the top to the bottom of the primitive structure are ${d}_{1}, {D}_{1}, {D}_{3}, {D}_{3}, {D}_{2}, {d}_{2}$, the heights of the corresponding cylinders are ${h}_{1}, {h}_{2}, {h}_{3}, {h}_{3}, {h}_{2}, {h}_{1}$, and satisfy the equivalent relationship of ${h}_{1}$+${h}_{2}$+${h}_{3}$=1/2*H*. For S3, the soft material is added to the middle position of the primitive, and the structural parameters are the same as S2. Here, we define the ratio of narrow diameter of soft materials as asymmetry degree:

1 $$\left\{\begin{array}{c}\frac{{d}_{2}}{{d}_{1}}={N}_{1} for S1\\ \frac{{d}_{2}}{{d}_{1}}={N}_{2} for S2\\ \frac{{D}_{2}}{{D}_{1}}={N}_{3} for S3\end{array}\right.$$

In order to facilitate comparative analysis, the hard and soft materials of three samples are polymers and silicone rubber, respectively. The material parameters are shown in Table 1.

**Table 1:** Material parameters.

Materials	*ρ* (kg/m^3^)	*Ε* (Gpa)	*υ*
Ploymer	1190	3	0.4
Silicon Rubber	1300	0.1175 × ${10}^{-3}$	0.47

**Figure 2:**
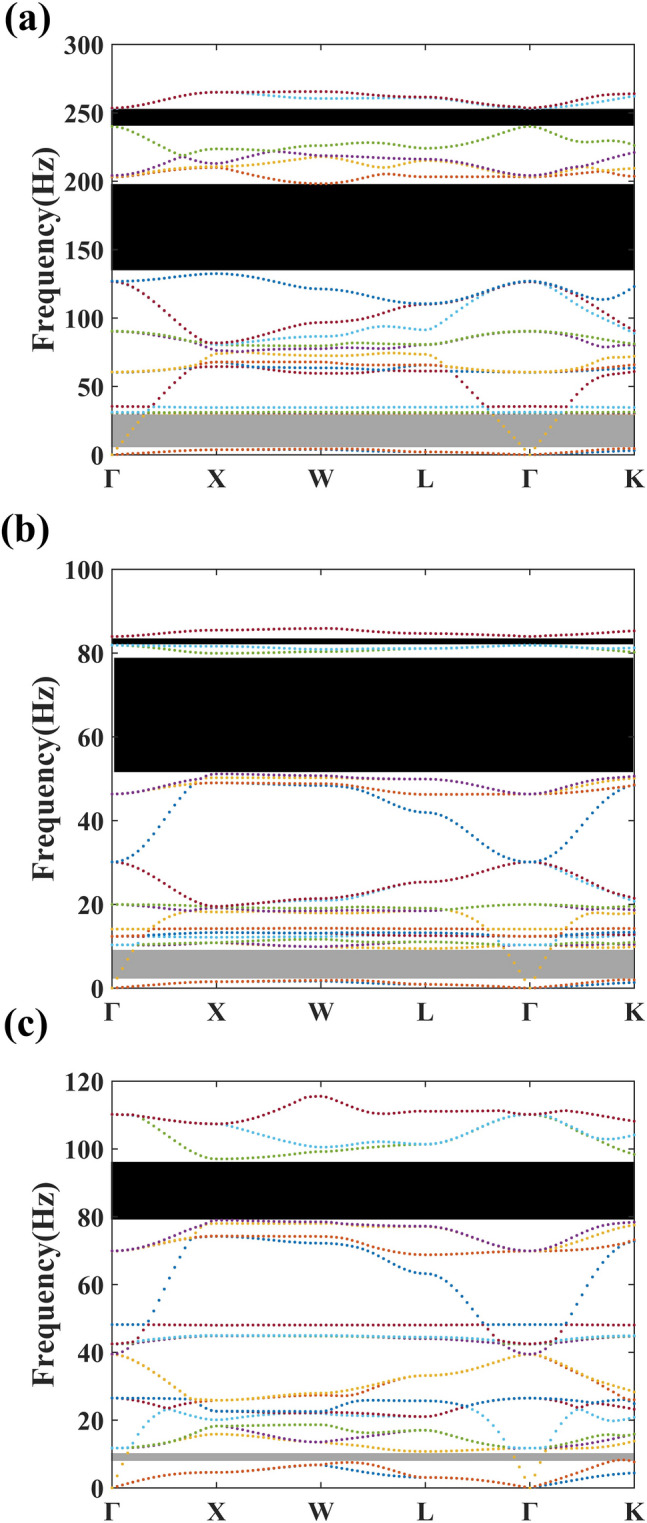
The phononic band structure of locally resonant PMs composed by (**a**) S1, (**b**) S2, (**c**) S3.

For calculating the phononic band structure, the Bloch boundary conditions are applied on the primitive unit cells of the three locally resonant PMs in the finite element simulation software COMSOL Multiphysics. The fixed structure parameters are S1: *a* = 37.3 mm, *H* = 16.15 mm,$D$=3 mm,${d}_{1}$=0.55 mm,${h}_{1}$=$0.1$ *H*. S2:${D}_{3}$=3 mm,${D}_{1}$=${D}_{2}$=1.5 mm,${d}_{1}$=0.55 mm,${h}_{1}$=$0.1$ *H*,${h}_{2}$=$0.3$ *H*,${h}_{3}$=$0.1$ *H*. S3:${D}_{3}$=3 mm,${D}_{1}$=1.5 mm,${d}_{1}$=${d}_{2}$=0.55 mm,${h}_{1}$=$0.1$ *H*,${h}_{2}$=$0.3$ *H*, ${h}_{3}$=$0.1$ *H*. Select the asymmetry degrees ${N}_{1}$=${N}_{2}$=${N}_{3}$=0.6 as a reference, and the calculated band structures of the three samples are shown in Fig. 2.

Existing pentamode metamaterials mainly consider cloak from external sound sources, that is, to study their single-mode properties (to realize decoupling of compressional and shear waves). However, it is equally important to study how to control noise inside acoustic cloaks, that is, the effect of structural parameters on the complete phononic band gap (PBG). In the PBG region, the acoustic waves that fall within the frequency range of the PBG emitted by the sound source inside the cloaked object are all confined inside the PMs, preventing the internal sound source from propagating outward. The phononic band structure of the S1 is shown in Fig. 2a. Not only are there two PBG in the phononic band structure diagram, but also the relatively flat energy bands appear near PBG. These flat energy bands mean the existence of resonance modes. The frequencies of the lower edge (${f}_{l}$) and upper egde (${f}_{u}$) of the first PBG are 132.42 Hz and 198.19 Hz, respectively. The relative bandwidth of the first PBG (Δω/${\omega }_{g}$ =$\frac{\Delta \omega }{({f}_{u}+{f}_{l})/2}$) is 0.398. The lower and upper edge frequencies of the second PBG are 240.06 Hz and 253 Hz, respectively. The relative bandwidth of the second PBG is 0.052. The phononic band structure of the S2 is shown in Fig. 2b. It can be seen that two complete PBGs can be opened in addition to the single-mode area. The lower and upper edge frequencies of the first PBG are 51.16 Hz and 79.95 Hz, respectively. The relative bandwidth of the first PBG is 0.439. The lower and upper edge frequencies of the second PBG are 81.9 Hz and 83.97 Hz, respectively. The relative bandwidth of the second PBG is 0.025. The phononic band structure of the S3 is shown in Fig. 2c. It is obvious that there is only one PBG in the phononic band structure diagram. The lower and upper edge frequencies of the first PBG are 78.99 Hz and 97.06 Hz, respectively. The relative bandwidth of the first PBG is 0.205.

From the above analysis, it can be seen that under the same structural parameters, the phononic band gap of S3 is narrower than S1 and S2, however, the stability of the S3 structure is higher. Elastic Stability of Stress rods was used to analyze the stability of three locally resonance PMs. Considering the length coefficient and the constraints of the rod, the three structures mentioned in this paper can be approximately equivalent to the structure of the pressure rod with fixed ends. The stability of the three structures is compared by calculating the critical force of the compression rod (${P}_{cr}$) respectively.

2 $$P_{cr} { = }\frac{{\pi^{2} EI}}{{(\mu l)^{2} }},\mu = 0.5,l = H$$

Here, *E* is the elastic model of the materials, *l* is the length of the rod, and *I* is the moment of inertia of the cross-section of the rod to the main axis of the row center.

Consider that the Young's modulus of soft materials is smaller than hard materials. And the deformation generally occurs in the silicone rubber part first. Here we only compare and analyze the corresponding stability of the soft material of the structures, and the obtained results are also applicable to the analysis of the hard material part.

For the three structures with the same structural and material parameters, only *I* differs in Eq. (2). And *I* can be expressed as $I = \frac{{\pi D^{4} }}{64}$ for the cross-section of the rod is a circle, *D* is the diameter of the section circle. Obviously, *I* in the formula ${P}_{cr}$ of S3 is larger than S1 and S2, so the ${P}_{cr}$ of S3 is larger than S1 and S2. Thus, the structure of S3 is more stable than S1 and S2.

To sum up, compared with the asymmetric double-cone locally resonant PMs, the two types multilayer cylindrical locally resonant PMs can not only obtain the complete PBG, also can greatly reduce the frequency of the first PBG. For S2 and S3, they can reduce the lower edge frequency of the first PBGs by 61.4% and 40.3%, respectively. In addition, the bandwidth of the first PBG can be extended, which is 10.3% higher than the relative bandwidth of asymmetric double-cone locally resonant PMs. This means that using locally resonant PMs formed by S2 to control ultra-low frequency acoustic/elastic waves will produce more excellent effects. However, it is clear that locally resonant PMs formed by S3 are more stable than S2.

## The influence of asymmetry degrees on the first PBG

**Figure 3:**
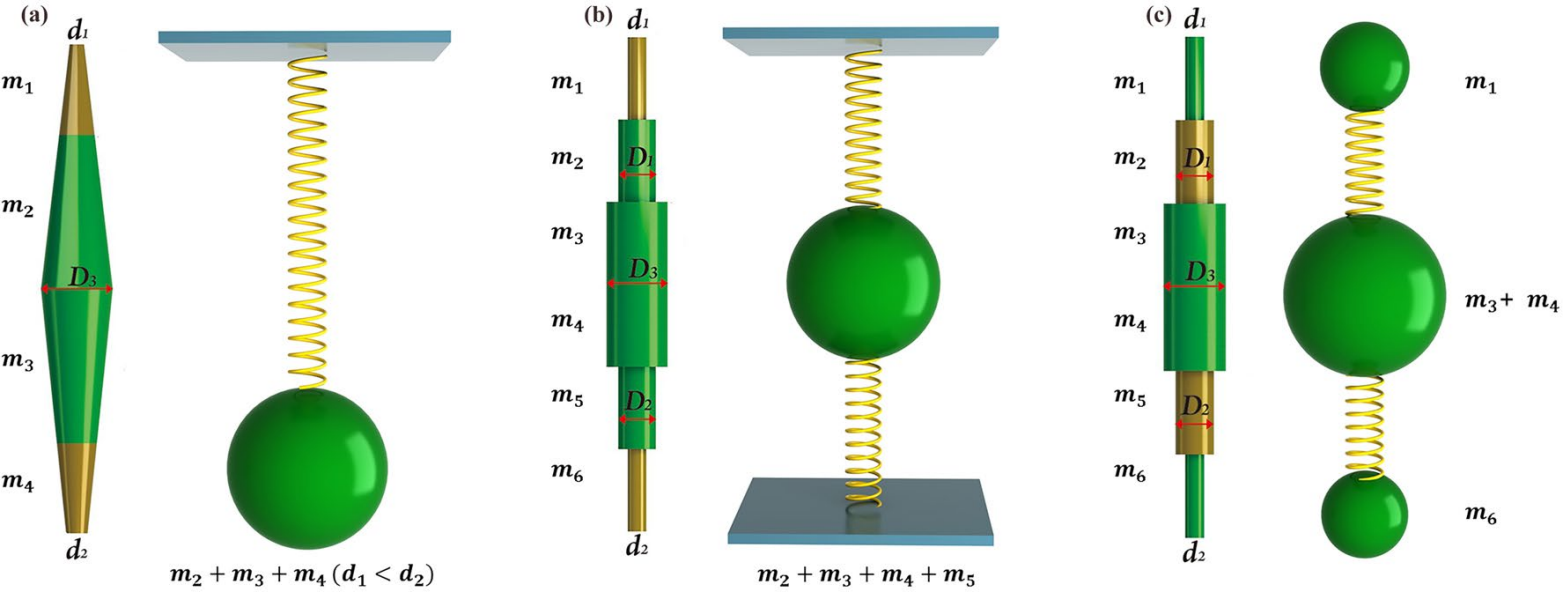
The model diagram of the “spring mass” system of the (**a**) S1, (**b**) S2 and (**c**) S3, respectively.

The Bragg scattering mechanism emphasizes the influence of periodic structure on waves, while the locally resonant mechanism emphasizes the interaction between the resonance characteristics of the scatterer unit and the waves in the matrix. Since the lower and upper frequencies of the PBG of the locally resonant PMs can be equivalent to the principle of the “spring-mass” system, by analyzing the equivalent parameters in the simplified model, the change trend of the PBG is analyzed. The model diagram of the “spring mass” system of the three structures is shown in Fig. 3. And the relationship between the lower edge frequency of the first PBG and the equivalent stiffness ${k}_{eff\_Si}$ and the equivalent mass ${M}_{eff\_Si}$ of the three types locally resonant PMs can be expressed in the following proportional relationship:

3 $${f}_{l\_Si}=\frac{1}{2\pi }\sqrt{\frac{{k}_{eff\_Si} }{{M}_{eff\_Si}}}(i=\mathrm{1,2},3)$$

At the same time, the equivalent stiffness ${k}_{eff\_Si}$ and equivalent mass ${M}_{eff\_Si}$ of three locally resonant PMs are approximately expressed as^23^:

4 $$\left\{\begin{array}{c}{k}_{eff\_S1}\propto \frac{{C}_{11}\left[{d}_{1}\left(H-2{h}_{1}\right)\right]+2{h}_{1}{D}_{3}}{H{h}_{1}} ({d}_{1}<{d}_{2})\\ {k}_{eff\_S1}\propto \frac{{C}_{11}\left[{d}_{2}\left(H-2{h}_{1}\right)\right]+2{h}_{1}{D}_{3}}{H{h}_{1}} \left({d}_{1}>{d}_{2}\right)\\ {M}_{eff\_S1}={m}_{2}+{m}_{3}+{m}_{4 }({d}_{1}<{d}_{2})\\ {M}_{eff\_S1}={m}_{1}+{m}_{2}+{m}_{3} ({d}_{1}>{d}_{2})\end{array}\right.\mathrm{for S}1$$

5 $$\left\{\begin{array}{l}{k}_{eff\_S2}\propto \frac{{C}_{11}({{d}_{1}}^{2}+{{d}_{2}}^{2})}{{h}_{1}}\\ {M}_{eff\_S2}={m}_{2}+{m}_{3}+{m}_{4}+{m}_{5}\end{array}\right.\mathrm{for S}2$$

6 $$\left\{\begin{array}{l}{k}_{eff\_S3}\propto \frac{{C}_{11}({{D}_{1}}^{2}+{{D}_{2}}^{2})}{{h}_{1}} \\ {M}_{eff\_S3}={{m}_{1}+m}_{2}+{m}_{3}+{m}_{4}+{\alpha }_{1}{m}_{5}+{m}_{6} ({D}_{1}<{D}_{2})\\ {M}_{eff\_S3}={m}_{1}+{m}_{3}+{m}_{4}+{m}_{6}+{\alpha }_{2}\left({m}_{2}+{m}_{5}\right) \left({D}_{1}>{D}_{2}\right)\\ 0<{\alpha }_{1}<\mathrm{1,0}<{\alpha }_{2}<1\end{array}\right. \mathrm{for S}3$$

where ${C}_{11}$=${\lambda }_{silicone rubber}$+$2{\mu }_{silicone rubber}$,${\lambda }_{silicone rubber}$=$6\times {10}^{-4}GPa$,${\mu }_{silicone rubber}$=$4\times {10}^{-5}GPa$. At the position of the soft materials, only part of the silicone rubber will deform, providing the elasticity required for structural deformation, while the other part provides the stiffness. Therefore, ${\alpha }_{1}$ and ${\alpha }_{2}$ are the undeformed coefficients at the position where the soft material is added.

**Figure 4:**
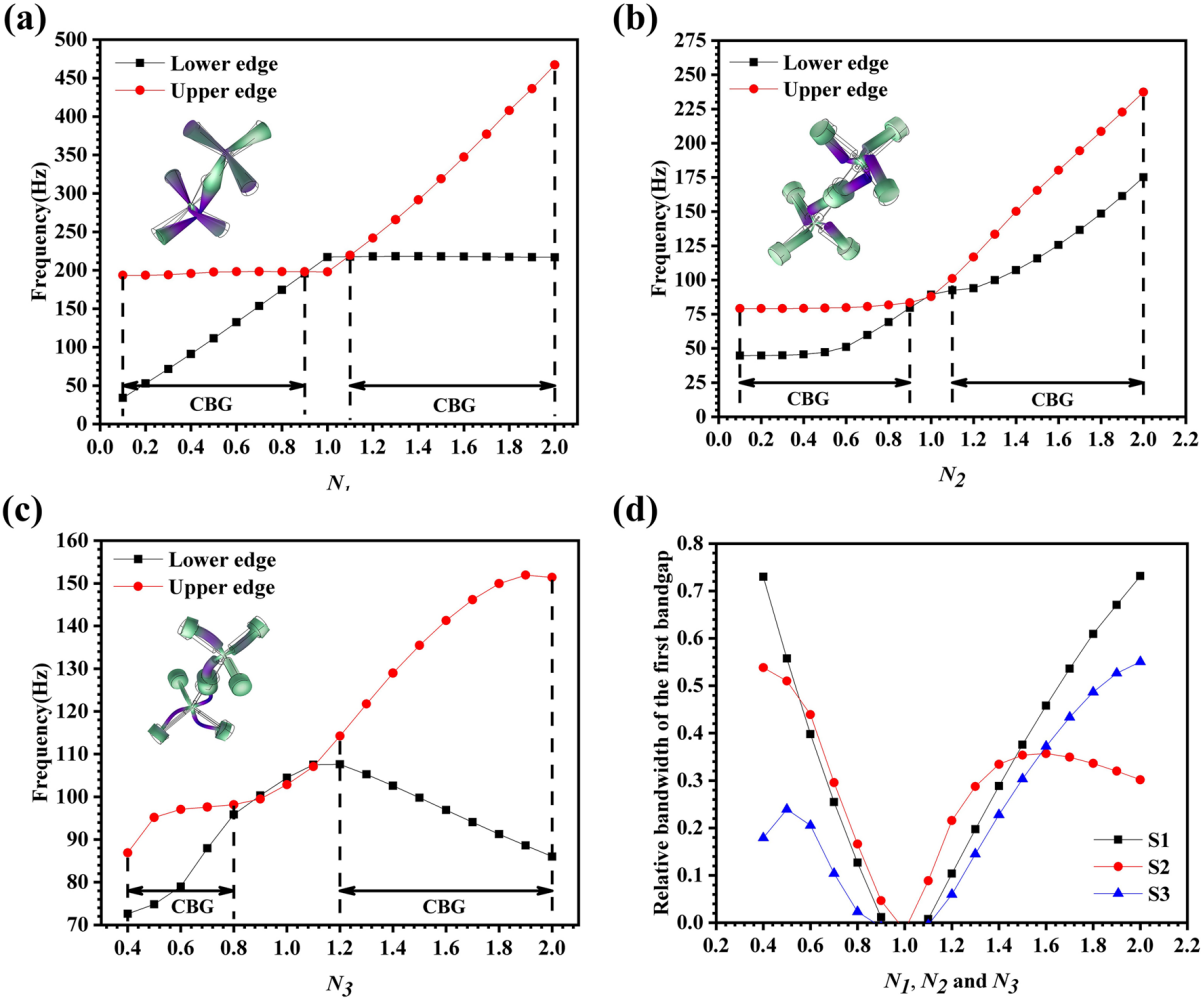
The influence of ${N}_{1}, {N}_{2}, {N}_{3}$ on the PBG of (**a**) S1, (**b**) S2 and (**c**) S3. (**d**) Comparison of the influence of asymmetry degrees on the relative bandwidth of the first PBG of three samples (S1, S2, S3).

Any structural parameter that can cause changes in the equivalent parameters of the locally resonant PMs will have an impact on the lower and upper frequencies of the first PBGs. In order to further study the locally resonant characteristics of the three samples, the influence of the asymmetry degrees (${N}_{1}$, ${N}_{2}$, and ${N}_{3}$) on the first PBG and relative bandwidth was studied and some results were shown in Fig. 4.

The influence of ${N}_{1}$ on the first PBGs of S1 was shown in Fig. 4a, and the vibration mode of the lower edge frequency of the first PBG is given when ${N}_{1}$ = 0.4. It can be seen that when ${N}_{1}$ ranges from 0.1 to 0.9, ${d}_{2}$ is more easily to deform. With the increase of ${N}_{1}$, the narrow diameter ${d}_{2}$ increases, the equivalent stiffness ${k}_{eff\_S1}$ produced by its deformation also is increased, while the change of the equivalent mass ${M}_{eff\_S1}$ is negligible, so the lower edge frequency of first PBG increases with increase of ${N}_{1}$. When ${N}_{1}$ ranges from 1.1 to 2.0, ${d}_{1}$ is more easily to deform. At this time, the lower edge frequency of the first PBG is mainly determined by the equivalent stiffness ${k}_{eff\_S1}$ produced by the deformation of ${d}_{1}$, while ${d}_{1}$ is a fixed parameter, which basically remains constant. Therefore, the lower edge frequency of the first PBG is basically unchanged.

The influence of ${N}_{2}$ on the first PBGs of S2 was shown in Fig. 4b, and the vibration mode of the lower edge frequency of the first PBG is given when ${N}_{2}$ = 0.4. When ${N}_{2}$ changes from 0.1 to 2.0, the equivalent stiffness ${k}_{eff\_S2}$ is determined by both ${d}_{1}$ and ${d}_{2}$ due to the deformation at both ${d}_{1}$ and ${d}_{2}$. With the increase of ${N}_{2}$, the equivalent stiffness ${k}_{eff\_S2}$ also increases, while the equivalent mass ${M}_{eff\_S2}$ remains basically unchanged, so the lower edge frequency of the first PBG increases with the increase of ${N}_{2}$.

The influence of ${N}_{3}$ on the first PBGs of S3 was shown in Fig. 4c, and the vibration mode of the lower edge frequency of the first PBG is given when ${N}_{3}$ = 0.4. When ${N}_{3}$ changes from 0.1 to 1.0, the equivalent stiffness ${k}_{eff\_S3}$ generated by the deformation at ${d}_{1}$ and ${d}_{2}$ increases at the same time, so the lower edge frequency of the first PBG gradually increases with the increase of ${N}_{3}$. When ${N}_{3}$ changes from 1.1 to 2.0, as ${d}_{1}$ and ${d}_{2}$ continue to increase, the deformation part of the soft material becomes smaller, and the part that does not deform increases, which causes the equivalent mass ${M}_{eff\_S3}$ increases. At the same time, since the equivalent stiffness ${k}_{eff\_S3}$ decreases, the lower edge frequency of the first PBG shows a downward trend as ${N}_{3}$ increases.

Figure 4d shows the influence of asymmetry degrees on the relative bandwidth of the first PBG of the three samples (S1, S2, S3). When the asymmetry degrees change in the range of 0.6–1.4, the relative bandwidth of the S2 is significantly higher than that of the S1. In addition, when ${N}_{1}$ and ${N}_{2}$ change in the range of 0.6–1.4, the lower edge frequency of the first PBG of S1 increased from 91.1 to 218.12 Hz, while the lower edge frequency of the first PBG of S2 increased from 51.16 to 107.2 Hz. Therefore, compared with the S1 type locally resonant PMs, the S2 type locally resonant PMs can not only reduce the lower edge frequency of the first PBG by 43.8–50.9%, but also broaden the relative bandwidth of the first PBG.

## Single-mode properties

**Figure 5:**
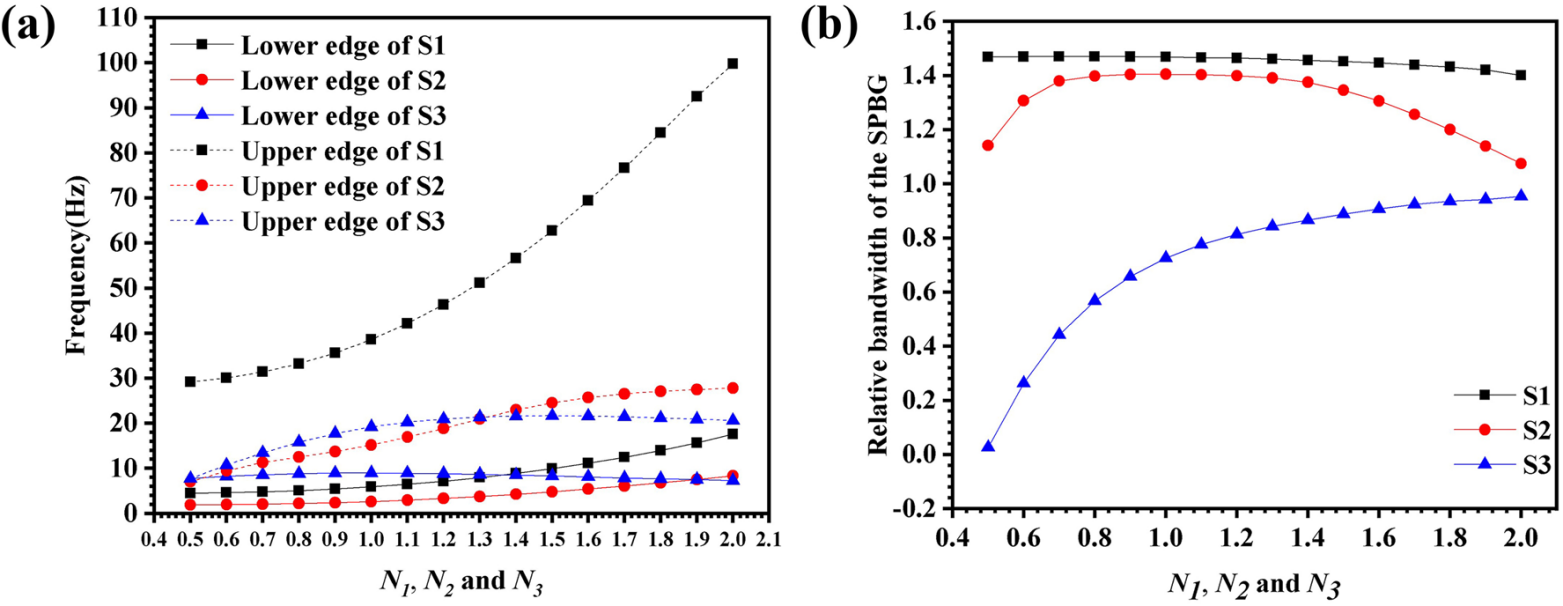
The influence of asymmetry degrees ${N}_{1}$, ${N}_{2}$, ${N}_{3}$ on the (**a**) upper and lower edge frequencies and (**b**) relative bandwidth of the single-mode areas.

The single-mode area is the frequency region that limits the decoupling of compression waves and shear waves, and it is an important factor to measure the pentamodal performance of locally resonant PMs, Therefore, it is necessary to further study the single-mode performance of the three samples. Figure 5 shows the effects of asymmetry degrees ${N}_{1}$, ${N}_{2}$, and ${N}_{3}$ on the upper and lower edge frequencies and relative bandwidths of the single-mode regions of the three samples. Obviously, the single-mode lower edge frequency of S1 and S2 increases slowly with the increase of asymmetry, and S3 shows a trend of first rising and then decreasing slightly with the increase of asymmetry. Among them, the single-mode lower edge frequency corresponding to S1 is the lowest. With the increase of asymmetry, the single-mode upper edge frequency of S1 and S2 showed a sharp increase and a gentle increase trend, respectively, while S3 first slowly increased and then decreased slightly. Therefore, the relative bandwidth of the single-mode areas of the S1 is the largest, which is basically maintained at about 1.46. while S2 is second, showing a trend of first rising and then decreasing. The relative bandwidth of the single-mode areas of S3 gradually increases with the increase of asymmetry ${N}_{3}$, and reaches the maximum value of 0.95 when ${N}_{3}$ is equal to 2.0.

## Pentamodal performance

The FOM is defined as the ratio of the equivalent bulk modulus (*B*) to the equivalent shear modulus (*G*). The larger the FOM, the easier it is to decouple the compression and shear waves, thereby obtaining good pentamodal performance. The FOM has the following proportional relationship with the ratio of compression and shear waves phase velocity:

7 $$\mathrm{FOM}=\frac{B}{G}\propto {\left(\frac{{C}_{B}}{{C}_{G}}\right)}^{2}$$

While, the compression wave phase velocity (${C}_{B}$) and shear wave phase velocity (${C}_{G}$) of locally resonant PMs are determined by the slope of the compression and shear waves of phononic band structure.

**Figure 6:**
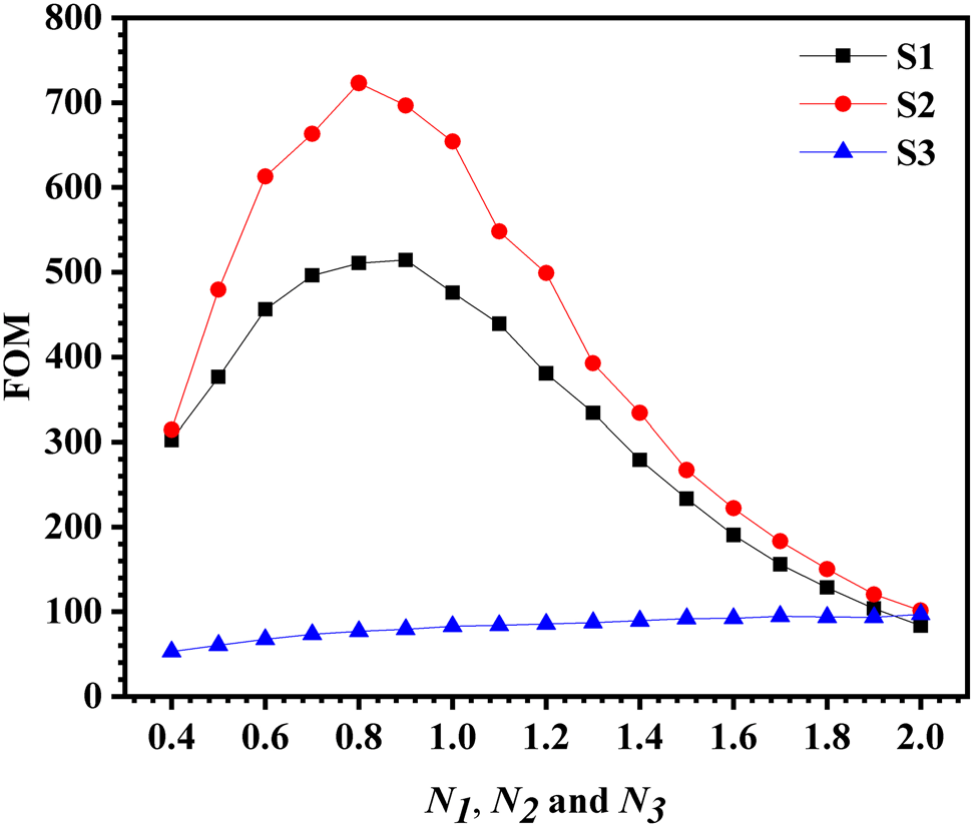
The influence of asymmetry degrees on FOM of the three samples.

Figure 6 shows the influence of asymmetry degrees on the FOM of three samples. Both S1 and S2 showed a trend of first increasing and then decreasing with the increase of asymmetry degrees. The FOM of S1 reached the maximum value of 514.29 when ${N}_{1}$ = 0.9, and the FOM of S2 reached the maximum value of 723.1 when ${N}_{2}$ = 0.8. For S3, it increases slowly with the increase of ${N}_{3}$, and FOM reaches the maximum value of 97.05 when ${N}_{3}$ = 2.0. The above analysis shows that, compared with S1, the FOM of S2 can be increased by up to 40.6%.

## Conclusion

For Bragg scattering PMs, using the PMs to achieve underwater acoustic insulation below 100 Hz requires a material with a several meters thick lattice size. In this paper, two multilayer composite cylindrical three-dimensional PMs are proposed. In order to compare and analyze with the asymmetrical double-cone locally resonant PMs, a large number of numerical calculations have been carried out on the influence of asymmetry degrees ${N}_{1}$, ${N}_{2}$, ${N}_{3}$ on the phononic band gap, single-mode properties, and FOM of the three samples (S1, S2, S3). Numerical results show that compared with S1 type locally resonant PMs, S2 type locally resonant PMs proposed in this paper can reduce the lower edge frequency of the first PBG by up to 61.4%, increase the relative bandwidth of the first PBG by up to 10.3%, and increase the FOM by up to 40.6%. This research will provide a theoretical basis and reference for further promoting the application of three-dimensional PMs in underwater low-frequency acoustic waves control devices.
